# The analysis of the progressive local failure process of the Longquan Reservoir dam based on the global-local dynamic strength reduction method

**DOI:** 10.1038/s41598-025-13298-4

**Published:** 2025-08-01

**Authors:** Xin Qu, Fangfang Diao, Cheng Li, Xingqian Xu, Zihan Wang, Shuai Hao

**Affiliations:** 1 School of Civil Engineering and Transportation, Anyang Institute of Technology, Anyang Henan, 455000 China; 2 School of Foreign Languages, Anyang Institute of Technology, Anyang Henan, 455000 China; 3 School of Architectural Engineering, Kaili University, Kaili, 556011 China; 4 School of Water Resources, Yunnan Agricultural University, Kunming Yunnan, 650201 China

**Keywords:** Physical degradation pattern, Strain softening, Strength reduction, Longquan reservoir dam, Local failure mechanism, Hydrogeology, Civil engineering, Natural hazards

## Abstract

During the progressive failure process of the slope (dam), the strength parameters of the soil in the slope (dam) continuously degrade, with the degree and rate of degradation of the soil near the shear zone significantly exceeding those in other areas. To address this mechanism, this paper proposes a global-local dynamic strength reduction method that simultaneously accounts for both the physical degradation pattern of the soil and the strain softening characteristics of the shear zone. Taking the Longquan Reservoir Dam as an engineering case, a two-dimensional profile calculation model at station 0 + 142 was established using ANSYS. Combining this model with the global-local dynamic strength reduction method, the progressive local failure process of the dam under heavy rain conditions was simulated. By analyzing the distribution patterns and evolution trends of dam displacement, stress, and plastic strain, the local failure mechanism of the dam was elucidated. The results indicate that the global-local dynamic strength reduction method can effectively captures both the soil’s physical degradation and the shear zone’s softening mechanism, accurately reflecting the failure progression of the dam while maintaining high computational efficiency. The critical strength reduction coefficients required to reach the critical instability state using different methods exceed 1.0, indicating that the Longquan Reservoir Dam is in a safe condition, which is consistent with field observations. The progressive local failure process of the dam shows that heavy rainfall induces persistent degradation of soil strength parameters. Initially, the downstream soil undergoes plastic yielding and shear flow. As the shear failure zone continues to expand, the soil from the mid-upstream shifts downstream, ultimately leading to the collapse at the dam crest and the formation of a continuous shear zone.

## Introduction

Since the 21 st century, the water conservancy engineering sector has experienced rapid development. The construction of landmark hydraulic projects, including the Three Gorges Project, Longtan Reservoir, Longyangxia Hydropower Station, and Xinanjiang Dam, has delivered substantial socioeconomic benefits to local communities. Concurrently, these megastructures have introduced complex geotechnical risks, elevating dam safety and stability to critical priorities in infrastructure governance^1–5^. In order to accurately assess dam safety and guide engineering construction, extensive research on reservoir dam stability has been conducted^6–17^. For example, Shi established stress calculation formulas based on the failure mechanism of soil slopes and used the finite element method to solve for the minimum stability factor of soil slopes under static conditions^6^. Li conducted static and seismic performance studies on the gravity dam of Guanmenzuizi Reservoir and optimized its cross-section dimensions using genetic algorithms^7^. Tang provided a comprehensive discussion of the geological characteristics and causes of Zhexi Reservoir, analyzing the variation characteristics of landslides under three different working conditions and specifically recommended reinforcing unstable slopes with anchor bars^8^. These studies confirm the effectiveness of finite element methods in dam safety evaluation.

Recent advances in dam safety analysis have yielded multiple innovative methodologies. Representative studies include: Sun et al. employed a rigorous three-dimensional limit equilibrium method to analyze the stability of the Wujiang landslide near the Wutou River Reservoir during the water storage process and identified the main sliding direction of the landslide^9^. Huang et al. integrated field investigations, displacement monitoring and numerical simulation to elucidate coupled influence mechanism of rainfall-reservoir interactions on the deformation characteristics and failure evolution of the Outlet Landslide^10^. Chen et al. applied the failure mode and effects analysis method (FMECA)-fuzzy analytic hierarchy process to the safety analysis of a certain reservoir dam in Yunnan Province, and the calculation process was consistent with the actual situation, confirming the effectiveness of the method^11^. Xiang et al. established a hybrid model combining support vector machines and fractal interpolation for reasonable predictive analysis of dam deformation^12^. While demonstrating significant domain-specific advantages, these sophisticated approaches demand specialized expertise that currently constrains widespread implementation.

Finite element method for reservoir dam stability assessment has now reached significant methodological maturity. Key applications demonstrate its capabilities: Yu employed ANSYS to establish a three-dimensional gravity dam model, analyzing the stress and strain fields under three different working conditions and concluding that the dam was in a stable state^13^. Xu et al. simulated the deformation evolution process of the Sanmendong landslide under the combined effects of reservoir water level changes and rainfall using ABAQUS, with results showing that the slope was in a stable state^14^. Wang analyzed the stress and displacement fields of the Niuerdong Reservoir dam under four working conditions (completion, design flood level, normal water storage level, and verification flood level) via ANSYS^15^. Meng et al. investigated pore pressure-seepage-parameter degradation relationships through a coupled finite element-limit equilibrium method, deriving dam safety factors^16^. Zang conducted ABAQUS-based seepage-stress coupled analysis at Shilihe Reservoir, establishing predictive equations for upstream slope safety factors during drawdown^17^. The aforementioned studies have significantly expanded the application of the finite element method in dam stability analysis. However, when determining the stability factor of the reservoir dam, the studies mentioned above have all employed the equal strength reduction method (traditional strength reduction method), which assumes that the strength parameters of the soil (cohesion, internal friction angle) are reduced equally. Research has found that during the deformation and failure process of the dam, the rates, degrees, and order of action of the reduction of cohesion and internal friction angle may vary significantly. Evidently, the equal strength reduction method cannot accurately reflect the true failure mechanism of dam.

To explore the different reduction mechanisms of cohesion and internal friction angle, many scholars have begun researching asynchronous strength reduction methods. Tang and Zheng were among the first to propose the double strength reduction method, suggesting that the reduction coefficient for cohesion is greater than that for the internal friction angle, and that both strength parameters are reduced according to a certain paired reduction ratio^18^. Chen et al. employed the dynamic strength reduction method to track progressively expanding slip surfaces. By integrating the advantages of global strength reduction for safety factor calculation, they computed dynamic safety factors throughout the slope’s progressive failure process, thereby achieving comprehensive analysis and control of the entire slope instability mechanism^19^. Xue et al. derived the non-proportional correlation between the reduction coefficients for cohesion and internal friction angle based on a linear decay assumption for strength parameter distribution. They incorporated this relationship into the finite element strength reduction method, resulting in the finite element strength parameter non-proportional correlation reduction method^20^. Xiao et al. analyzed extensive experimental data on cohesive soil slopes to establish statistical laws describing the decay of cohesion and internal friction angle under varying water content. Based on this, they proposed a double reduction coefficient strength reduction method that reflects the natural degradation of geotechnical mechanical parameters, along with a comprehensive safety coefficient calculation method^21^. Zhu et al. introduced the concept of a strength reserve area and derived an expression for the slope safety factor using the double strength reduction method, highlighting essential differences caused by different virtual initial points that lead to two distinct reduction paths^22^. Zhang et al. considered the reduction effects of changes in water content on the cohesion and internal friction angle of red clay, proposing a double strength reduction method based on these influences^23^.

The aforementioned studies focus on either the physical degradation of soil strength parameters or the strain softening characteristics of shear zones, providing partial insights into the instability mechanisms of slopes (dams). However, there remains an important issue unresolved. During the progressive failure process of the slope (dam), the rates and magnitudes of the degradation of soil strength parameters at different parts of the slope (dam) may vary slightly, with the degree and rate of degradation of the soil near the shear zone manifesting significantly more than other areas. How to effectively incorporate these spatial differences in the degradation mechanisms of soil strength parameters warrants further investigation. Based on the conclusions of previous studies, this paper proposes a global-local dynamic strength reduction method that simultaneously accounts for both the physical degradation of geotechnical strength parameters and the strain-softening behavior of the shear zone. Taking the Longquan Reservoir dam as an engineering case, we establish a two-dimensional profile calculation model of the dam using ANSYS to simulate the progressive local failure process. Through the analysis of deformation, stress, and plastic strain distributions and their evolution, the study reveals the local failure mechanism of the dam.

## Global-Local dynamic strength reduction method

The safety stability factor of slopes (dams) is determined using the finite element strength reduction method. The basic principle involves iteratively reducing strength parameters, such as cohesion *c* and *φ*, until the slope (dam) reaches a critical instability state. This method does not require predefined assumptions about the shape or location of the sliding surface, making it widely applicable^24,25^. Since geological materials possess both cohesive strength and frictional strength, employing a single reduction coefficient in dam stability analysis fails to accurately capture the differing rates and extents of degradation in cohesion and internal friction angle^26,27^. Therefore, this paper employs a double strength parameter reduction coefficient method to investigate their respective strength safety reserves, with the calculation formulas as follows:

1 $${c_e}=\frac{c}{{{F_c}}},{\kern 1pt} {\kern 1pt} \tan{\varphi _e}=\frac{{\tan\varphi }}{{{F_\varphi }}}$$

where *c*_*e*_ is the reduced cohesion; *φ*_*e*_ is the reduced internal friction angle; *F*_*c*_ is the cohesion reduction coefficient; and *F*_*φ*_ is the internal friction angle reduction coefficient. When *F*_*c*_= *F*_*φ*_, this corresponds to the traditional strength reduction method. Generally, the degree and rate of degradation in cohesion are greater than those of the internal friction angle.

### Double strength parameter reduction mechanism

During the progressive failure process of the slope (dam), the rates and magnitudes of degradation of geotechnical strength parameters at different parts of the slope (dam) may vary slightly, with the degradation degree and rate of the soil near the shear zone manifesting significantly more than other areas. Therefore, this paper combines with the local and global reduction methods to simulate the varying degradation patterns of soil strength parameters in different parts of the slope (dam). The process of reducing strength parameters is not arbitrarily set but should follow relevant physical and mechanical laws. Rainfall infiltration increases the moisture content of the soil, gradually weakening the bonds among bound and capillary water molecules, which leads to a reduction in cohesion. Additionally, increased moisture expands the spacing between soil particles, decreasing the relative rolling, sliding, and interlocking forces among them, thereby reducing the internal friction angle. Before sliding occurs, a shear zone must develop within the slope, which is closely related to the soil’s strain-softening behavior. Clearly, rainfall infiltration causes a general degradation of soil strength parameters, a behavior typical of all soils, while the local strain-softening characteristics are confined specifically to the shear zone vicinity. Thereby, this paper considers that the global reduction mechanism primarily stems from moisture-induced decreases in cohesion and internal friction angle, while the local reduction mechanism is governed by the strain-softening characteristics within the shear zone.

### Impact of moisture content on soil shear strength

The degradation pattern of soil mechanical parameters due to changes in moisture content is the foundational principle for the global reduction of strength parameters in this study. Wang fitted an exponential function to obtain the relationship of reduction coefficients for soil strength parameters^28^, as shown in Table 1.

**Table 1:** Relationship of reduction coefficients for strength parameters of unsaturated soil^26^.

Type	Fitting Formula	*R* ^2^	Reduction Relationshi
Hefei Expansive Soil	*C* = 270.425*e*^−0.0771*w*^ tan*φ* = 0.948*e*^−0.948*w*^	0.9817 0.9754	*F*_*φ*_ *= F*_*c*_^*0.576*^
Xiaolangdi Landslide	*C* = 46.261*e*^−0.04172*w*^	0.9953	*F*_*φ*_ *= F*_*c*_^*0.423*^
	tan*φ* = 0.551*e*^−0.01764*w*^	0.9573	
Sanmenxia Landslide	*C* = 142.820*e*^−0.04388*w*^	0.9922	*F*_*φ*_ *= F*_*c*_^*0.793*^
	tan*φ* = 0.838*e*^−0.0348*w*^	0.9458	
Changsha Red Clay	*C* = 314.402*e*^−0.11338*w*^	0.9086	*F*_*φ*_ *= F*_*c*_^*0.958*^
	tan*φ* = 1.671*e*^−0.10863*w*^	0.8927	

Based on the fitted data, he proposed a functional relationship between the reduction coefficients of the two strength parameters^28^, expressed in Eq. (2):

2 $${F_\varphi }={F_c}^{k}$$

When *k* = 1, this corresponds to the traditional strength reduction method; when *k* = 0, it represents the traditional overload safety coefficient. Since the degradation rate of cohesion *c* exceeds that of the internal friction angle, this paper adopts Wang’s suggested value of *k* = 0.5 as the corresponding reduction relationship for the two shear strength parameters.

### Strain softening characteristics of shear zone

**Fig. 1:**
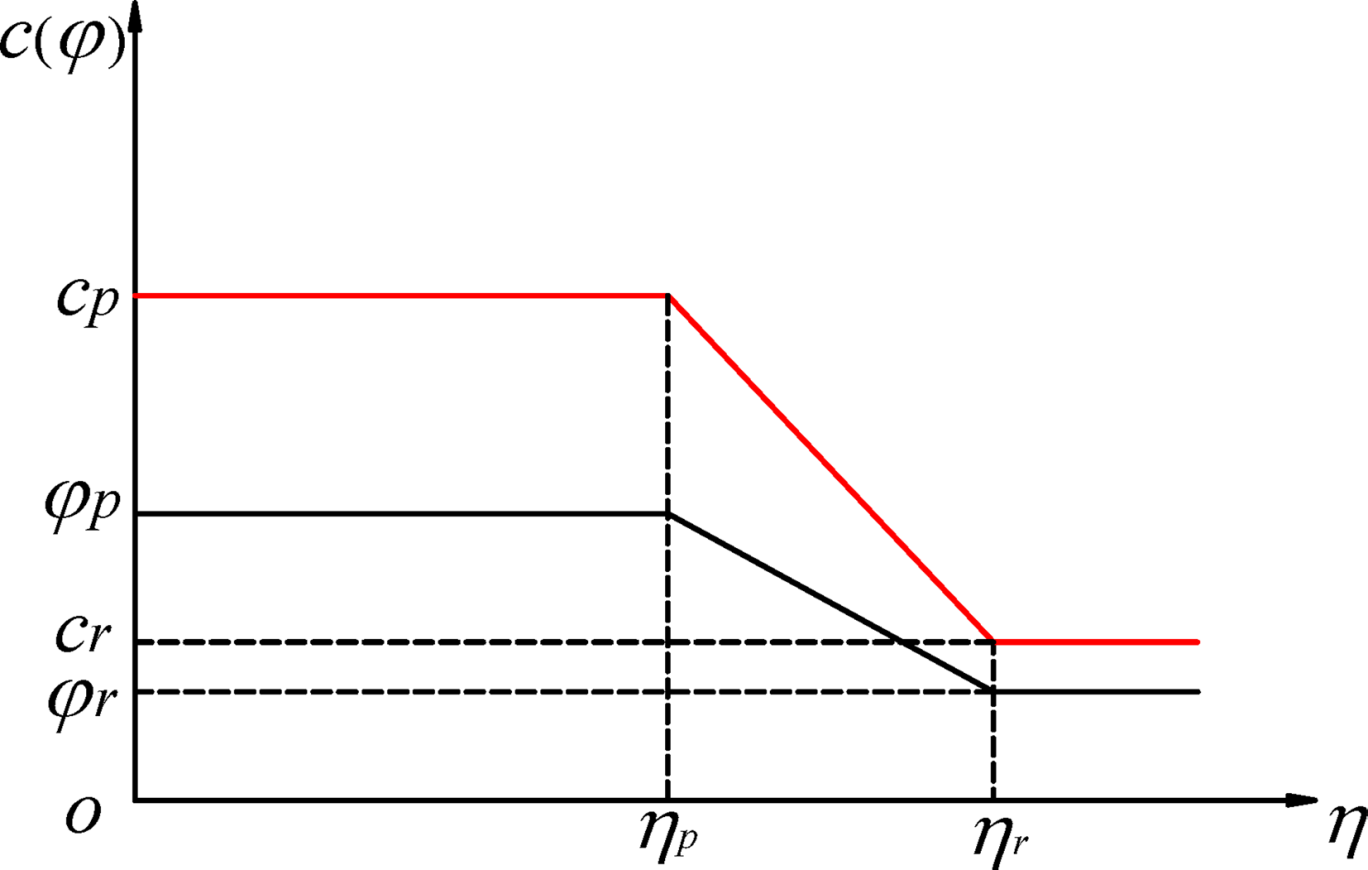
Schematic diagram of the strain softening model.

The linear strain softening constitutive model in FLAC3D is used to simulate the strain softening characteristics of shear zone. In this model, the strength parameters change linearly with the softening parameters, as illustrated in Fig. 1, with the specific functional expressions as follows:

3 $$c(\eta )=\left\{ \begin{gathered} {c_p}{\kern 1pt} {\kern 1pt} {\kern 1pt} {\kern 1pt} {\kern 1pt} {\kern 1pt} {\kern 1pt} {\kern 1pt} {\kern 1pt} {\kern 1pt} {\kern 1pt} {\kern 1pt} {\kern 1pt} {\kern 1pt} {\kern 1pt} {\kern 1pt} {\kern 1pt} {\kern 1pt} {\kern 1pt} {\kern 1pt} {\kern 1pt} {\kern 1pt} {\kern 1pt} {\kern 1pt} {\kern 1pt} {\kern 1pt} {\kern 1pt} {\kern 1pt} {\kern 1pt} {\kern 1pt} {\kern 1pt} {\kern 1pt} {\kern 1pt} {\kern 1pt} {\kern 1pt} {\kern 1pt} {\kern 1pt} {\kern 1pt} {\kern 1pt} {\kern 1pt} {\kern 1pt} {\kern 1pt} {\kern 1pt} {\kern 1pt} {\kern 1pt} {\kern 1pt} {\kern 1pt} {\kern 1pt} {\kern 1pt} {\kern 1pt} {\kern 1pt} {\kern 1pt} {\kern 1pt} {\kern 1pt} {\kern 1pt} {\kern 1pt} {\kern 1pt} {\kern 1pt} {\kern 1pt} {\kern 1pt} {\kern 1pt} {\kern 1pt} {\kern 1pt} {\kern 1pt} {\kern 1pt} {\kern 1pt} {\kern 1pt} {\kern 1pt} {\kern 1pt} {\kern 1pt} {\kern 1pt} {\kern 1pt} {\kern 1pt} {\kern 1pt} {\kern 1pt} {\kern 1pt} {\kern 1pt} {\kern 1pt} {\kern 1pt} {\kern 1pt} {\kern 1pt} {\kern 1pt} {\kern 1pt} {\kern 1pt} {\kern 1pt} {\kern 1pt} {\kern 1pt} {\kern 1pt} {\kern 1pt} {\kern 1pt} {\kern 1pt} {\kern 1pt} {\kern 1pt} (\eta \leqslant {\eta _p}) \hfill \\ {c_p}+\frac{{\eta - {\eta _p}}}{{{\eta _r} - {\eta _p}}}({c_r} - {c_p}){\kern 1pt} {\kern 1pt} {\kern 1pt} {\kern 1pt} {\kern 1pt} {\kern 1pt} {\kern 1pt} {\kern 1pt} {\kern 1pt} {\kern 1pt} {\kern 1pt} {\kern 1pt} {\kern 1pt} {\kern 1pt} {\kern 1pt} {\kern 1pt} {\kern 1pt} {\kern 1pt} {\kern 1pt} ({\eta _p}<\eta <{\eta _r}) \hfill \\ {c_r}{\kern 1pt} {\kern 1pt} {\kern 1pt} {\kern 1pt} {\kern 1pt} {\kern 1pt} {\kern 1pt} {\kern 1pt} {\kern 1pt} {\kern 1pt} {\kern 1pt} {\kern 1pt} {\kern 1pt} {\kern 1pt} {\kern 1pt} {\kern 1pt} {\kern 1pt} {\kern 1pt} {\kern 1pt} {\kern 1pt} {\kern 1pt} {\kern 1pt} {\kern 1pt} {\kern 1pt} {\kern 1pt} {\kern 1pt} {\kern 1pt} {\kern 1pt} {\kern 1pt} {\kern 1pt} {\kern 1pt} {\kern 1pt} {\kern 1pt} {\kern 1pt} {\kern 1pt} {\kern 1pt} {\kern 1pt} {\kern 1pt} {\kern 1pt} {\kern 1pt} {\kern 1pt} {\kern 1pt} {\kern 1pt} {\kern 1pt} {\kern 1pt} {\kern 1pt} {\kern 1pt} {\kern 1pt} {\kern 1pt} {\kern 1pt} {\kern 1pt} {\kern 1pt} {\kern 1pt} {\kern 1pt} {\kern 1pt} {\kern 1pt} {\kern 1pt} {\kern 1pt} {\kern 1pt} {\kern 1pt} {\kern 1pt} {\kern 1pt} {\kern 1pt} {\kern 1pt} {\kern 1pt} {\kern 1pt} {\kern 1pt} {\kern 1pt} {\kern 1pt} {\kern 1pt} {\kern 1pt} {\kern 1pt} {\kern 1pt} {\kern 1pt} {\kern 1pt} {\kern 1pt} {\kern 1pt} {\kern 1pt} {\kern 1pt} {\kern 1pt} {\kern 1pt} {\kern 1pt} {\kern 1pt} {\kern 1pt} {\kern 1pt} {\kern 1pt} {\kern 1pt} {\kern 1pt} {\kern 1pt} {\kern 1pt} {\kern 1pt} {\kern 1pt} {\kern 1pt} {\kern 1pt} {\kern 1pt} {\kern 1pt} (\eta \geqslant {\eta _r}) \hfill \\ \end{gathered} \right.$$

4 $$\varphi (\eta )=\left\{ \begin{gathered} {\varphi _p}{\kern 1pt} {\kern 1pt} {\kern 1pt} {\kern 1pt} {\kern 1pt} {\kern 1pt} {\kern 1pt} {\kern 1pt} {\kern 1pt} {\kern 1pt} {\kern 1pt} {\kern 1pt} {\kern 1pt} {\kern 1pt} {\kern 1pt} {\kern 1pt} {\kern 1pt} {\kern 1pt} {\kern 1pt} {\kern 1pt} {\kern 1pt} {\kern 1pt} {\kern 1pt} {\kern 1pt} {\kern 1pt} {\kern 1pt} {\kern 1pt} {\kern 1pt} {\kern 1pt} {\kern 1pt} {\kern 1pt} {\kern 1pt} {\kern 1pt} {\kern 1pt} {\kern 1pt} {\kern 1pt} {\kern 1pt} {\kern 1pt} {\kern 1pt} {\kern 1pt} {\kern 1pt} {\kern 1pt} {\kern 1pt} {\kern 1pt} {\kern 1pt} {\kern 1pt} {\kern 1pt} {\kern 1pt} {\kern 1pt} {\kern 1pt} {\kern 1pt} {\kern 1pt} {\kern 1pt} {\kern 1pt} {\kern 1pt} {\kern 1pt} {\kern 1pt} {\kern 1pt} {\kern 1pt} {\kern 1pt} {\kern 1pt} {\kern 1pt} {\kern 1pt} {\kern 1pt} {\kern 1pt} {\kern 1pt} {\kern 1pt} {\kern 1pt} {\kern 1pt} {\kern 1pt} {\kern 1pt} {\kern 1pt} {\kern 1pt} {\kern 1pt} {\kern 1pt} {\kern 1pt} {\kern 1pt} {\kern 1pt} {\kern 1pt} {\kern 1pt} {\kern 1pt} {\kern 1pt} {\kern 1pt} {\kern 1pt} {\kern 1pt} {\kern 1pt} {\kern 1pt} {\kern 1pt} {\kern 1pt} {\kern 1pt} {\kern 1pt} {\kern 1pt} {\kern 1pt} {\kern 1pt} {\kern 1pt} (\eta \leqslant {\eta _p}) \hfill \\ {\varphi _p}+\frac{{\eta - {\eta _p}}}{{{\eta _r} - {\eta _p}}}({\varphi _r} - {\varphi _p}){\kern 1pt} {\kern 1pt} {\kern 1pt} {\kern 1pt} {\kern 1pt} {\kern 1pt} {\kern 1pt} {\kern 1pt} {\kern 1pt} {\kern 1pt} {\kern 1pt} {\kern 1pt} {\kern 1pt} {\kern 1pt} {\kern 1pt} {\kern 1pt} {\kern 1pt} ({\eta _p}<\eta <{\eta _r}) \hfill \\ {\varphi _r}{\kern 1pt} {\kern 1pt} {\kern 1pt} {\kern 1pt} {\kern 1pt} {\kern 1pt} {\kern 1pt} {\kern 1pt} {\kern 1pt} {\kern 1pt} {\kern 1pt} {\kern 1pt} {\kern 1pt} {\kern 1pt} {\kern 1pt} {\kern 1pt} {\kern 1pt} {\kern 1pt} {\kern 1pt} {\kern 1pt} {\kern 1pt} {\kern 1pt} {\kern 1pt} {\kern 1pt} {\kern 1pt} {\kern 1pt} {\kern 1pt} {\kern 1pt} {\kern 1pt} {\kern 1pt} {\kern 1pt} {\kern 1pt} {\kern 1pt} {\kern 1pt} {\kern 1pt} {\kern 1pt} {\kern 1pt} {\kern 1pt} {\kern 1pt} {\kern 1pt} {\kern 1pt} {\kern 1pt} {\kern 1pt} {\kern 1pt} {\kern 1pt} {\kern 1pt} {\kern 1pt} {\kern 1pt} {\kern 1pt} {\kern 1pt} {\kern 1pt} {\kern 1pt} {\kern 1pt} {\kern 1pt} {\kern 1pt} {\kern 1pt} {\kern 1pt} {\kern 1pt} {\kern 1pt} {\kern 1pt} {\kern 1pt} {\kern 1pt} {\kern 1pt} {\kern 1pt} {\kern 1pt} {\kern 1pt} {\kern 1pt} {\kern 1pt} {\kern 1pt} {\kern 1pt} {\kern 1pt} {\kern 1pt} {\kern 1pt} {\kern 1pt} {\kern 1pt} {\kern 1pt} {\kern 1pt} {\kern 1pt} {\kern 1pt} {\kern 1pt} {\kern 1pt} {\kern 1pt} {\kern 1pt} {\kern 1pt} {\kern 1pt} {\kern 1pt} {\kern 1pt} {\kern 1pt} {\kern 1pt} {\kern 1pt} {\kern 1pt} {\kern 1pt} {\kern 1pt} {\kern 1pt} {\kern 1pt} {\kern 1pt} {\kern 1pt} (\eta \geqslant {\eta _r}) \hfill \\ \end{gathered} \right.$$

where *c*_*p*_ is the peak cohesion; *c*_*r*_ is the residual cohesion; *φ*_*p*_ is the peak internal friction angle; *φ*_*r*_ is the residual internal friction angle; *η*_*p*_ is the post-peak softening parameter; *η*_*r*_ is the residual softening parameter. Commonly used softening parameters include equivalent plastic strain, maximum plastic principal strain, and plastic shear strain. Based on the linear strain softening model, the strength parameters *c* and *φ* can be expressed with the following relationship:

5 $$\frac{{c - {c_p}}}{{{c_r} - {c_p}}}=\frac{{\varphi - {\varphi _p}}}{{{\varphi _r} - {\varphi _p}}}$$

6 $$c={c_p}+\frac{{\varphi - {\varphi _p}}}{{{\varphi _r} - {\varphi _p}}}({c_r} - {c_p})$$

During the calculation, the degradation strength parameter *φ* can be obtained through Eq. (1), and the degradation decaying strength parameter *c* can be calculated using Eq. (6).

### Yield criteria

**Fig. 2:**
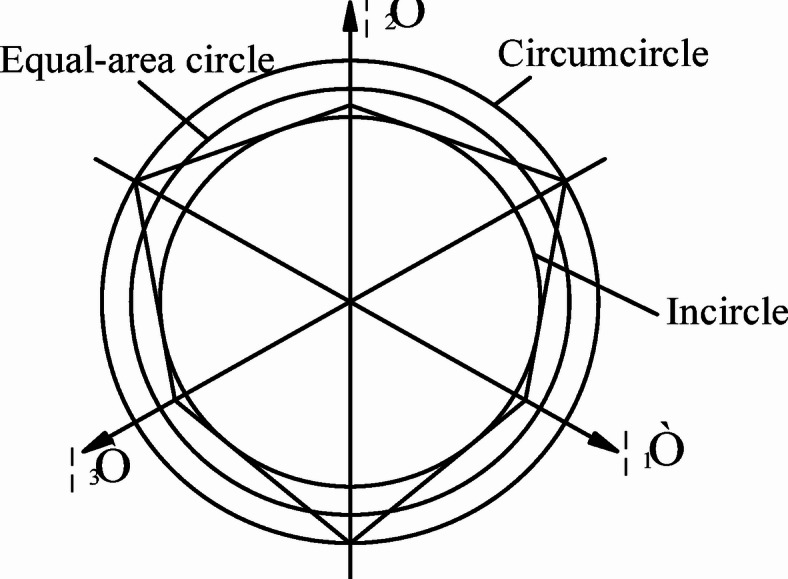
Yield curves with different α and k on the π-plane.

The Mohr-Coulomb (M-C) criterion does not account for the effect of the intermediate principal stress, and the yield surface is an irregular hexagonal cone, which appears as an irregular hexagon on the π plane (Fig. 2), characterized by sharp peaks and edges. This presents significant challenges in numerical calculations, and the sharp points can lead to singularities that prevent convergence of the results. When analyzing the dam stability using ANSYS, the Drucker-Prager (D-P) criterion is employed instead. The D-P criterion employs an ideal elastoplastic constitutive model known for its robust applicability^29^.

The functional expression of the D-P criterion is shown in Eq. (7):

7 $$F=a{I_1}+\sqrt {{J_2}} =k$$

Where,

8 $${I_1}={\sigma _1}+{\sigma _2}+{\sigma _3},{\kern 1pt} {\kern 1pt} {\kern 1pt} {\kern 1pt} {\kern 1pt} {J_2}=\frac{1}{6}[{({\sigma _1} - {\sigma _2})^2}+{({\sigma _1} - {\sigma _3})^2}+{({\sigma _2} - {\sigma _3})^2}]{\kern 1pt} {\kern 1pt} {\kern 1pt}$$

In Eq. (8), *I*_1_​ is the first invariant of the stress tensor, and *J*_2_​ is the second invariant of the deviatoric stress tensor.

Equation (7) is a general expression for the D-P criterion. To use different yield criteria in finite element analysis, it is necessary to continuously adjust the values of *α* and *k*.

(1) When *α* and *k* have the expression:

9 $$a=\frac{{2\sin \varphi }}{{\sqrt 3 (3 - \sin \varphi )}},{\kern 1pt} {\kern 1pt} {\kern 1pt} k=\frac{{6c\cos \varphi }}{{\sqrt 3 (3 - \sin \varphi )}}$$

At this stage, the yield surface on the π-plane becomes the circumcircle of an irregular hexagon, representing the default D-P criterion in ANSYS.

(2) When *α* and *k* take another expression:

10 $$a=\frac{{\sin \varphi }}{{\sqrt {3(3+{{\sin }^2}\varphi )} }},{\kern 1pt} {\kern 1pt} {\kern 1pt} k=\frac{{3c\cos \varphi }}{{\sqrt {3(3+{{\sin }^2}\varphi )} }}$$

At this stage, the yield surface on the π-plane corresponds to the circumcircle of the hexagon, matching the plane strain limit of the M-C criterion as represented by the D-P criterion.

During parameter input, data conversion is required through Eq. (11):

11 $${\varphi _2}=\arcsin \frac{{3\sin {\varphi _1}}}{{\sin {\varphi _1}+2\sqrt {(3+{{\sin }^2}{\varphi _1})} }},{\kern 1pt} {\kern 1pt} {\kern 1pt} {c_2}={c_1} \times \frac{{\tan {\varphi _2}}}{{\tan {\varphi _1}}}$$

### Dynamic plastic zone

The failure of a slope (dam) initiates at a specific location near the toe and progressively propagates upward. During this process, localized damage continuously expands, evolving into a distinct shear zone. Accurately identifying these damaged areas is therefore critical for analyzing progressive slope instability. In this study, the plastic zone of the slope is visualized by plotting regions where the ratio of nodal trial stress (not the actual stress) to yield surface stress exceeds 1.0. Regions with a ratio below 1.0 represent the elastic zone. The *c*-*φ* parameter framework of the Mohr-Coulomb criterion directly quantifies the shear strength mechanism of soils and demonstrates wider applicability in analyses of shallow-depth soil failure, safety factor calculations based on limit equilibrium methods, and assessments of tensile-shear composite failure. Therefore, to assess computational rationality, the M-C criterion (Eq. (12)) is implemented via a defined element table. This directly evaluates the yield state of each element, identifying the plastic zone satisfying the M-C criterion.

12 $$\frac{{{\sigma _1} - {\sigma _3}}}{2}=\frac{{{\sigma _1}+{\sigma _3}}}{2}\sin \varphi +c \cdot \cos \varphi$$

### Computational steps

The specific computational steps for the global-local dynamic strength reduction method are as follows:

(1) Set F*φ* = 1.1 and *Fc* = 1.21, reducing the strength parameters of the soil based on Eq. (1) to obtain the initial reduced strength parameters *φ*_1_, *c*_1_. Use Eq. (11) to calculate the required input parameter values *φ*_11_, *c*_11_.

(2) Perform slope (dam) simulation in ANSYS, calculating the stresses of each element and determining the plastic zone of the slope.

(3) Apply *Fφ* = 1.1 to reduce the angle of internal friction of the plastic zone elements using Eq. (1), yielding *φ*_2_. Calculate the cohesion *c*_2_ for these elements according to Eq. (6).

(4) Repeat steps (1), (2), and (3) until any two of the three criteria for slope instability failure are satisfied:

- Numerical non-convergence;
- Sudden, significant displacement change at key monitoring points;
- Full development of a continuous shear failure surface.

## Benchmark model analysis

### Homogeneous soil slope

**Fig. 3:**
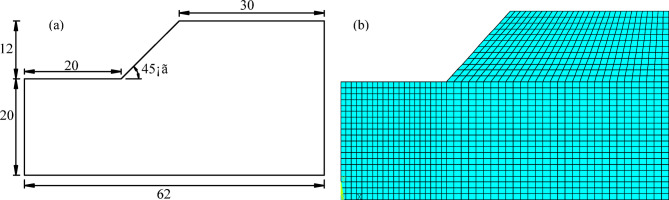
Schematic diagram of the slope model (units: m): **(a)** geometric model; **(b)** computational model.

The study^29^ found that when the distance from the slope toe to the left boundary exceeds 1.5 H (H is the slope height) the slope height, and the distance from the slope crest to the right boundary exceeds 2.5 H, with the total height of the upper and lower boundaries not less than 2 H, the calculation results are relatively ideal. Therefore, this paper adopts the benchmark case from reference^30^: the slope height is 12 m, the slope angle is 45º, the distance from the toe to the front edge of the model is 20 m, the distance from the top to the rear edge of the model is 30 m, the total height of the model is 32 m, and the total width is 62 m, as shown in Fig. 3(a). The soil material is silty clay, with properties derived from previous experimental data, as shown in Table 2. The computational model was divided into 4245 nodes and 1360 elements, as shown in Fig. 3(b). Boundary conditions: normal constraints were applied to both sides of the model, while the bottom was fully constrained.

**Table 2:** Slope calculation Parameters^30^.

*E*/MPa	*µ*	ρ/kg/m³	*c*_*p*_/kPa	*c*_cp._/kPa	*φ*_*p*_/°	*φ*_cp._/°	c_*r*_/kPa	*c*_cr_/kPa	*φ*_*r*_/°°	*φ*_cr_/°
56.5	0.4	1930	25	18.87	20	15.36	7.9	6.13	16.8	13.19

Note: *E* denotes elastic modulus; *µ* denotes poisson’s ratio; *ρ* denotes density; *c*_*p*_ denotes peak cohesion; *c*_*cp.*_ denotes converted peak cohesion; *φ*_*p*_ denotes peak internal friction angle; *φ*_*cp.*_ denotes converted peak internal friction angle; *c*_*r*_ denotes residual cohesion; *φ*_*cr*_ denotes converted residual cohesion; *φ*_*r*_ denotes residual internal friction angle; *φ*_*cr*_ denotes converted residual internal friction angle.

### Calculation scheme

To validate the accuracy and applicability of the proposed method, seven computational approaches were compared: the traditional strength reduction method (simultaneous *c*-*φ* reduction), *c*-only reduction method, *φ*-only reduction method, non-proportional reduction method (*Fc*/*Fφ* = constant), the double strength reduction Method 1 (global reduction method via formula (2), which considers the global reduction mechanism, neglecting the local strain-softening characteristics of shear zone), the double strength reduction Method 2 (local dynamic reduction method via formula (6), which accounts for the local strain-softening characteristics of shear zone, neglecting the global reduction mechanism), and the double strength reduction Method 3 (global-local dynamic strength reduction method via formulas (2) and (6), which can simultaneously capture the physical degradation laws of the strength parameters of soil and the softening mechanism of shear zone).

### Analysis of calculation results

**Fig. 4:**
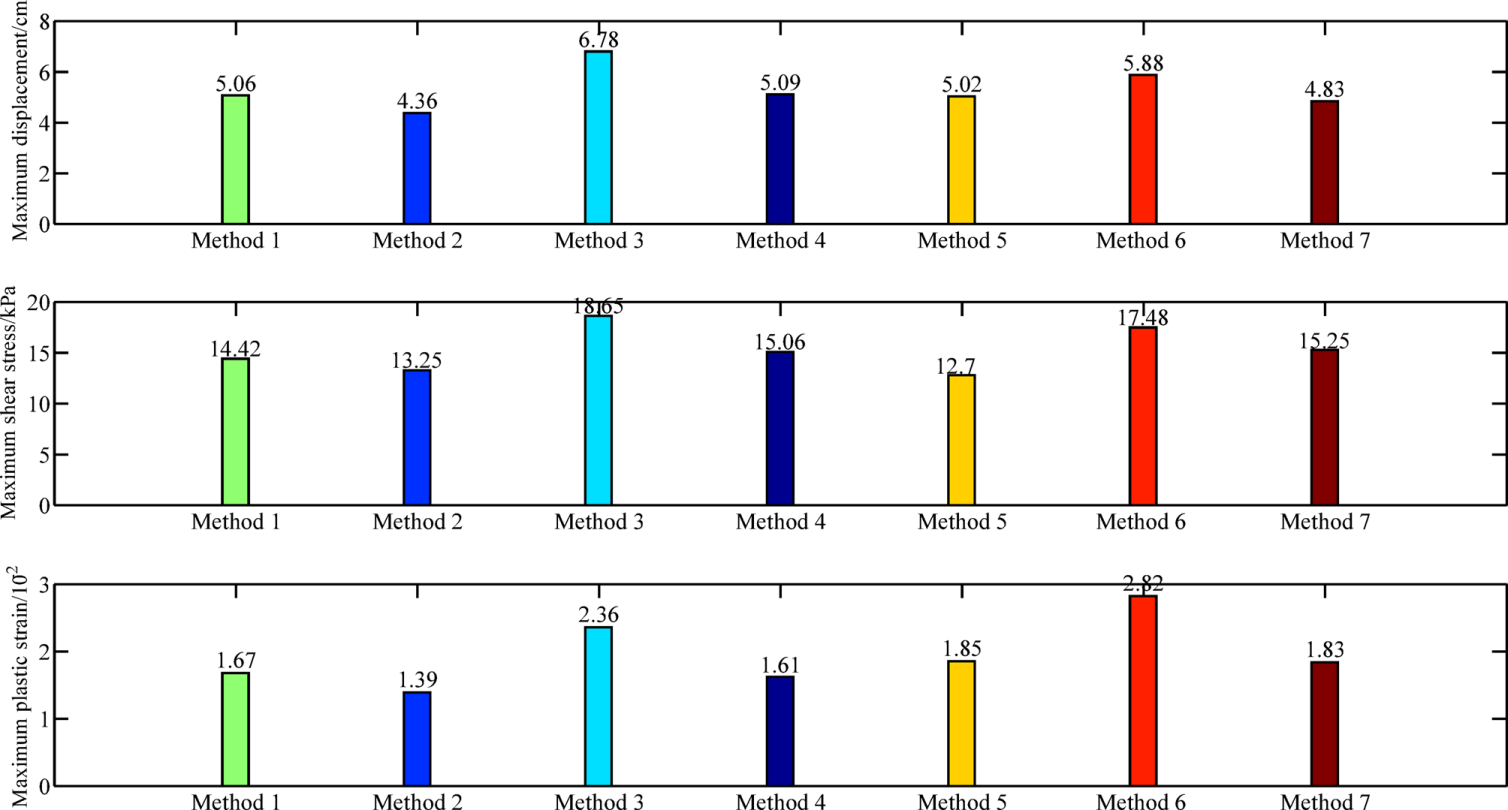
Comparison of maximum horizontal displacement, maximum shear stress, and maximum plastic strain of the slope obtained by different methods. Note: Method 1 denotes the traditional reduction method; Method 2 denotes *c*-only reduction method; Method 3 denotes *ϕ*-only reduction method; Method 4 denotes the non-proportional reduction method; Method 5 denotes the double strength reduction Method 1; Method 6 denotes the double strength reduction Method 2; Method 7 denotes the double strength reduction Method 3.

**Fig. 5:**
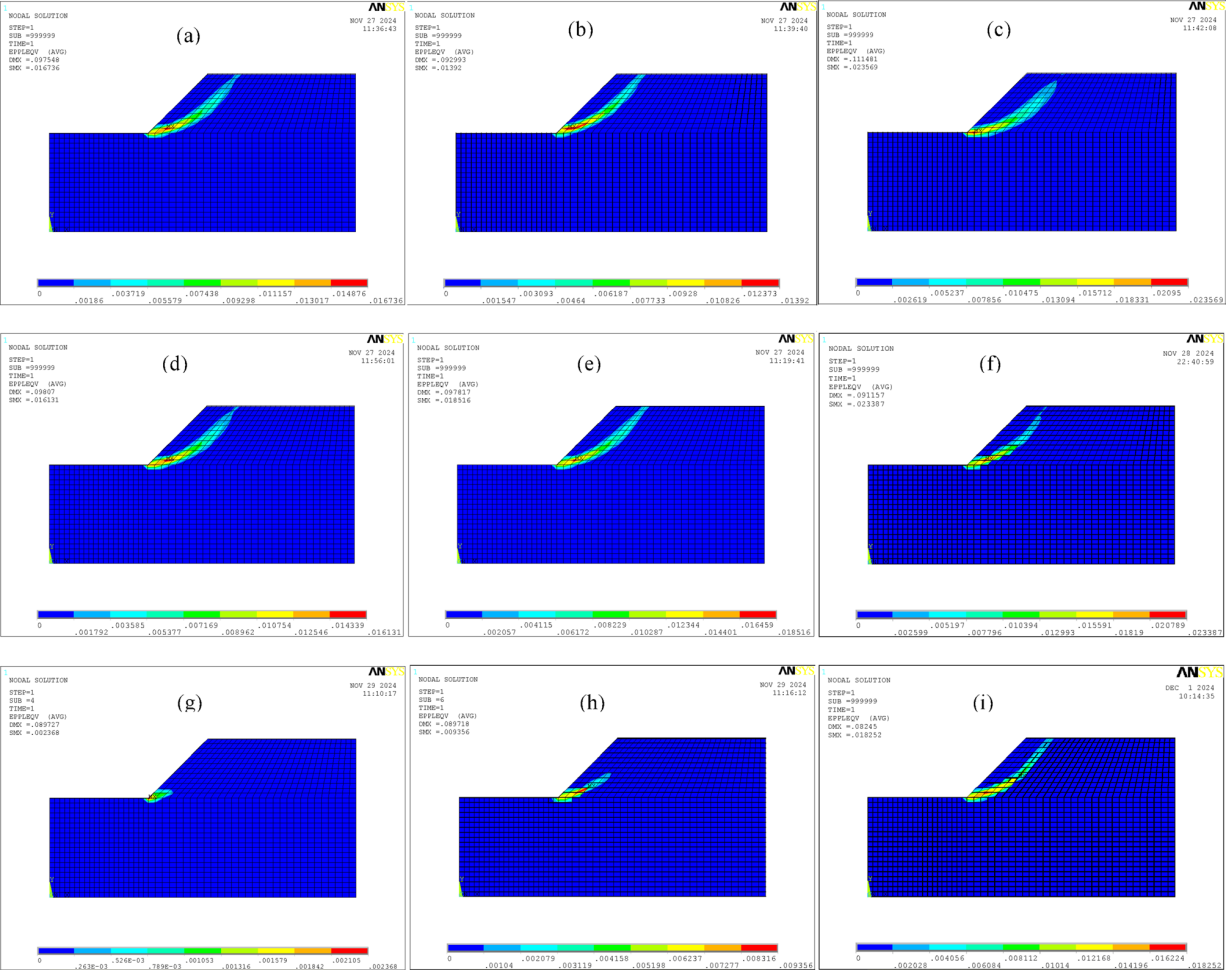
Plastic zones of the slope obtained by different methods: **(a)** traditional reduction method (*Fc* = *Fφ* = 1.27); **(b)** *c-*only reduction method (*Fc* = 1.63); **(c)** *φ-*only reduction method (*Fφ* = 1.73); **(d)** non-proportional reduction method (*Fc* = 1.23, *Fφ* = 1.3); **(e)** the double strength reduction method 1 (*Fc* = 1.37, *Fφ* = 1.17); **(f)** the double strength reduction method 2 (*Fφ* = 1.15); **(g)** original strength parameters; **(h)** the double strength reduction method 3 (global zone *Fc* = 1.1, *Fφ* = 1.05, shear zone *Fφ* = 1.05); **(i)** double strength reduction method 3 (global zone *Fc* = 1.21, *Fφ* = 1.1, shear zone *Fφ* = 1.05).

 The critical reduction coefficients, maximum horizontal displacement, maximum shear stress, and maximum plastic strain obtained using different reduction methods are shown in Table 3; Fig. 4, and Fig. 5.

The traditional strength reduction method and the non-proportional reduction method, as well-established approaches for slope stability analysis, have long been validated by numerous scholars regarding the reasonableness of their results^18,20,22,25,29,30^, despite their somewhat inadequate explanation of slope failure mechanism. The safety factor calculated by the limit equilibrium method is 1.32^20,30^, while the results obtained from the traditional strength reduction method and the non-proportional reduction method are both 1.27. The difference between the two sets of results is only 3.8%, which further validates the reasonableness of the outcomes derived from the traditional strength reduction method and the non-proportional reduction method. As shown in Fig. 4, the method only reduces the internal friction angle or local dynamic reduction method leads to significant horizontal displacements, shear stresses, and plastic strains. In contrast, the method only reduces the cohesion yields substantially smaller values. When geotechnical strength degradation laws are considered without accounting for shear zone softening mechanisms, computed shear stresses using the double strength reduction method 1 fall below expected levels. The calculation results of the traditional strength reduction method, non-proportional reduction method, and the global-local dynamic reduction method (the double strength reduction method 3) show close agreement, indicating the effectiveness of the double strength reduction method 3.

**Table 3:** Key values of slope calculated by different methods.

Different methods	Method 1	Method 2	Method 3	Method 4	Method 5	Method 6	Method 7
Critical reduction coefficient	***Fc =*** 1.27 ***Fφ*** **=** 1.27	***Fc =*** 1.63	***Fφ*** = 1.73	***Fc*** = 1.23 ***Fφ*** = 1.3	***Fc*** = 1.37 ***Fφ*** = 1.17	***Fφ*** = 1.15	***Fc =*** 1.21***Fφ*** = 1.1 (global zone) ***Fφ*** = 1.06 (shear zone)
Horizontal maximum displacement/cm	5.06	4.36	6.78	5.09	5.02	5.88	4.83
Maximum shear stress/kPa	14.42	13.25	18.65	15.06	12.7	17.48	15.25
Maximum plastic strain/10^−2^	1.67	1.39	2.36	1.61	1.85	2.82	1.83

Note: The critical reduction coefficient represents the strength reduction required to reach the critical instability state of the slope.

Figure 5 demonstrates that larger critical-state friction angles correlate with shear bands closer to the slope surface. This occurs because higher *φ*-values increase the critical shear force required for slope failure, resulting in steeper shear surfaces and more superficial localization. Figures 5(g), 5(h), and 5(i) illustrate the evolution process of the plastic zone in the slope simulated using the double strength reduction method 3: the plastic zone initially develops at the slope toe and progressively propagates upward to the crest.

## Analysis of engineering examples

### Overview of Longquan reservoir dam

**Fig. 6:**
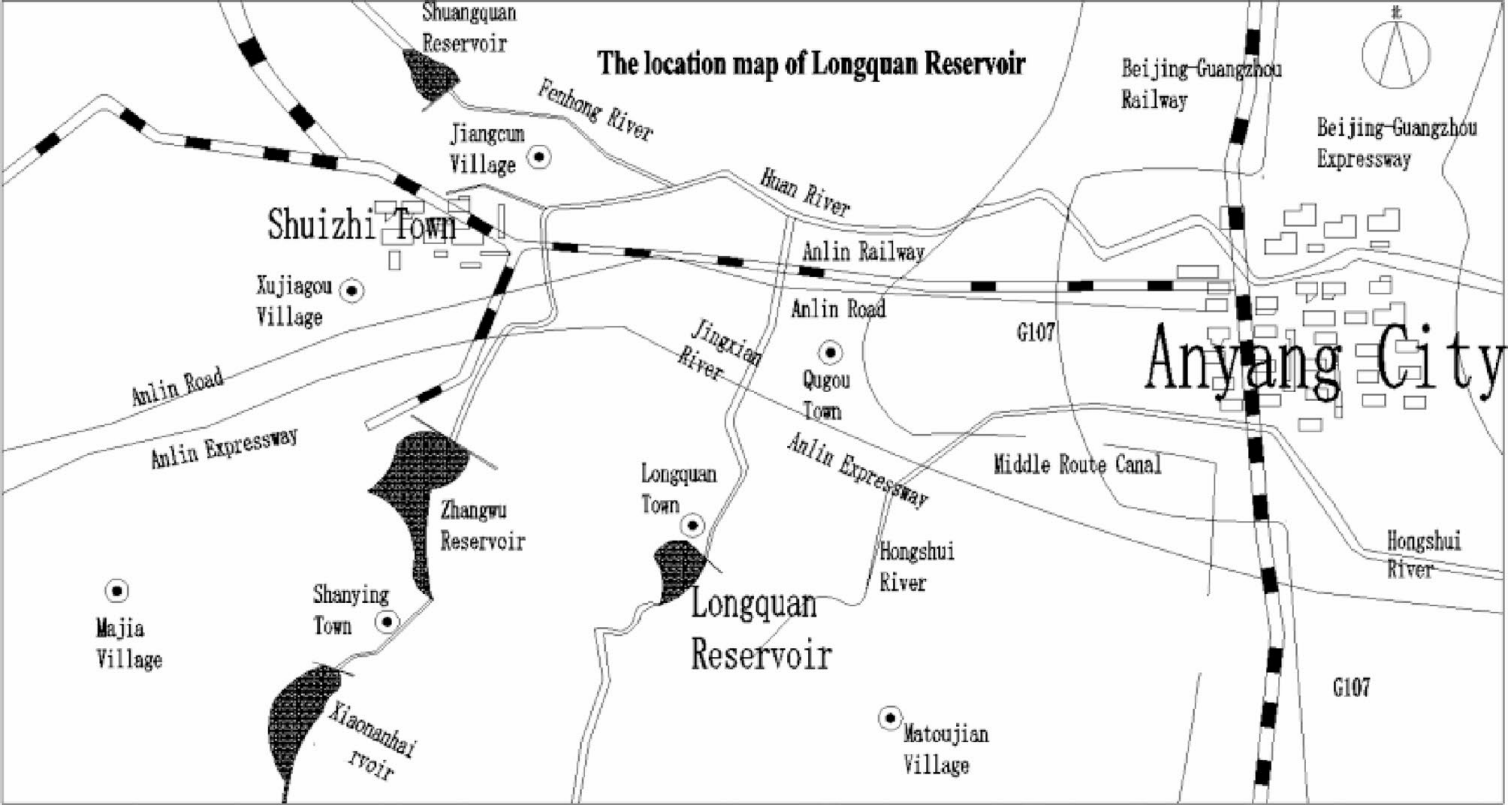
The location map of Longquan Reservoir.

As illustrated in Fig. 6, Longquan Reservoir is located 1 km southwest of Longquan Town in the Long’an District of Anyang City. It lies on the Jinxi River, a tributary of the Huan River in the Haihe River Basin. It is 18 km from the Anyang urban area, with a controlled catchment area of 32km^2^ and a total storage capacity of 2.83 million m^3^. The reservoir primarily serves flood control and also integrates functions such as irrigation and aquaculture. The hydraulic infrastructure of the reservoir comprises three primary components: the dam, spillway, and water conveyance tunnel. Downstream from the reservoir are Longquan Town and five natural villages, as well as the urban area of Anyang, the Central Route of the South-to-North Water Diversion Project, Anlin Expressway, Jingguang Railway, and National Highway 107. This proximity underscores the reservoir’s strategic geographical significance.

**Fig. 7:**
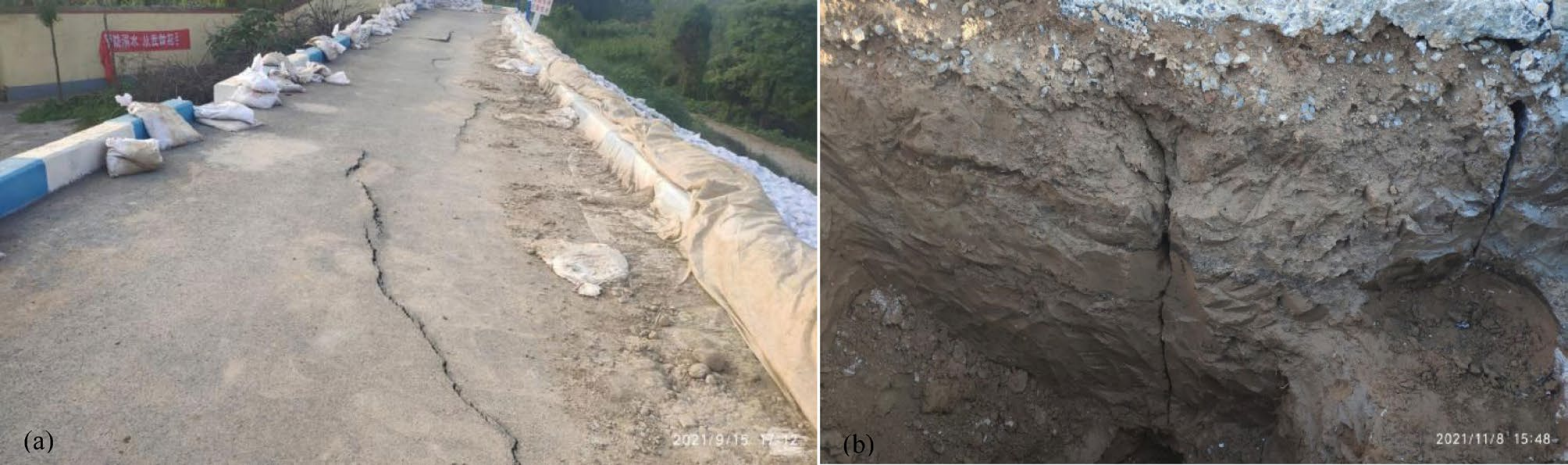
On-site damage photos: **(a)** crack and uneven settlement of dam crest; **(b)** crack on the south elevation of the trench (the right side is the upstream side).

From July 19 to 23, 2021, Anyang City experienced extreme rainfall, leading to a rapid rise in the reservoir’s water level to approximately 143.5 m. This triggered spillway discharge, peaking at 465 m³/s. The intense and prolonged rainfall caused substantial damage to the reservoir’s structures. Cracks and uneven settlement were observed on the crest, while the downstream dam exhibited deformation and bulging. The drainage ditch suffered compression and deformation. Erosion and damage occurred on both sides of the spillway’s masonry stone slope, and the masonry stone retaining wall deformed and collapsed, with severe erosion at the downstream dry stone section. A total of five cracks were identified on the dam crest, located at station coordinates 0 + 130 to 0 + 208, 0 + 227 to 0 + 248, 0 + 279 to 0 + 284, 0 + 304 to 0 + 339, and 0 + 360 to 0 + 391. The surface cracks ranged from 0.5 to 4 cm in width, while the internal cracks in the dam’s soil measured between 1 and 20 mm in width, as shown in Fig. 7(a). Preliminary assessments attribute the cracks to the reservoir’s original construction during the 1960–1970 s, when technical and material limitations led to inconsistent construction quality. Subsequent reinforcements and height increases further introduced structural discrepancies. Additionally, the passage of vehicles and pedestrians accelerated the development of the cracks. A test trench excavated near the main dam at 0 + 142 (Fig. 7(b)) exposed a layer of plastic heavy silty soil with signs of layered compaction and localized organic content. Probing data (N10) indicated high moisture content below a depth of 4 m, accompanied by uneven strength distribution. Four vertical cracks were observed on the trench’s sidewalls and base, measuring 1–20 mm in width and extending beyond 2 m depth. These cracks exhibited near-vertical development, forming an arch-like pattern centered on the downstream side.

**Fig. 8:**
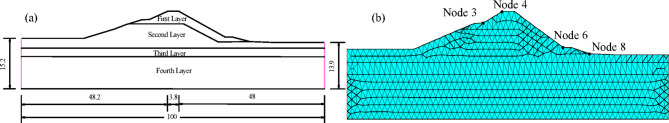
Pile No.0 + 142 dam (units: m) **(a)** profile model; **(b)** computational model.

The significant vertical cracks near station 0 + 142 pose a serious threat to the dam’s structural integrity, particularly under future heavy rainfall scenarios, raising concerns about localized instability. To evaluate this risk, a detailed profile model was developed for station 0 + 142 (Fig. 8(a)), incorporating the four geotechnical layers. First Layer: Gray-yellow medium silty soil, non-reactive to shaking, slightly glossy, with moderate dry strength and toughness. Local variations include heavy and light silty soil. Second Layer: Dark yellow plastic heavy silty soil, occasionally soft plastic, with minor inclusions of brick fragments and gravel. Third Layer: Gray-yellow medium silty soil, moderately reactive to shaking, non-glossy, with low dry strength and toughness. Fourth Layer: Brown-yellow plastic to hard plastic heavy silty soil, featuring rust-yellow oxidation spots and moderate geotechnical properties. The dam crest measures 3.8 m in width with an elevation of 147.3 m, while the upstream dam with a slope ratio of 1:3 is protected by C20 concrete.

A two-dimensional profile model for station 0+142 was established using ANSYS, employing PLANE82 element and a plane strain model^29,31,32^. The model was divided into 2490 nodes and 1175 elements, as shown in Figure 8(b). As indicated in Figure 8(b), the black dots mark monitoring nodes: nodes 3 and 4 on the upstream dam crest, and nodes 6 and 8 on the downstream face. Boundary conditions: normal constraints were applied to both sides of the model, while the bottom was fully constrained. The calculation parameters for the dam provided by Henan Yubei Water Resources Survey and Design Institute CO., LTD. are presented in Table 4.

**Table 4:** Calculation parameter of the dam.

Soil Layer	E/MPa	µ	ρ/kg/m³	c_*p*_/kPa	φ_*p*_/°	c_cp._/kPa	φ_cp._/°	c_*r*_/kPa	φ_*r*_/°	c_cr_/kPa	φ_cr_/°
First Layer	10	0.3	1900	16.2	16.4	12.5	12.9	4.05	10.3	3.3	8.42
Second Layer	7	0.35	1900	14.1	11.4	11.3	9.26	3.53	7.09	2.94	5.91
Third Layer	9	0.33	1970	15.1	15.9	11.7	12.55	3.78	9.97	3.09	8.17
Fourth Layer	11	0.3	1970	22.3	14.4	17.4	11.48	5.58	9.01	4.59	7.43

Note: *E* denotes elastic modulus; *µ* denotes poisson’s ratio; *ρ* denotes density; *c*_*p*_ denotes peak cohesion; *c*_*cp.*_ denotes converted peak cohesion; *φ*_*p*_ denotes peak internal friction angle; *φ*_*cp.*_ denotes converted peak internal friction angle; *c*_*r*_ denotes residual cohesion; *φ*_*cr*_ denotes converted residual cohesion; *φ*_*r*_ denotes residual internal friction angle; *φ*_*cr*_ denotes converted residual internal friction angle.

### Analysis of the progressive local failure process of the dam

To investigate the impact of heavy rainfall on the stability of the dam section at station 0 + 142 and its evolutionary failure process, this study employs three double strength reduction methods to analyze the displacement field and distribution characteristics of the plastic zone under self-weight effects, as well as the evolution trends. The displacements and stresses at monitoring points are recorded to explore the deformation evolution mechanism of the dam. The softening effect of heavy rain on the strength parameters of the soil is simulated by reducing the strength parameters. During each reduction calculation, the strength reduction coefficients for each soil layer are set to the same values, as shown in Table 5.

**Table 5:** Reduction values of strength parameters for three double strength reduction methods.

Different Methods		Strength Reduction Coefficient	First Layer	Second Layer	Third Layer	Fourth Layer
Double Strength Reduction Method 1		*Fc* = 1.21 *Fφ* = 1.1	*c* = 10.33kPa *φ* = 11.76°	*c* = 9.34kPa *φ* = 8.43°	*c* = 9.67kPa *φ* = 11.44°	*c* = 14.38kPa *φ* = 10.46°
		*Fc* = 1.44 *Fφ* = 1.2	*c* = 8.68kPa *φ* = 10.81°	*c* = 7.85kPa *φ* = 7.74°	*c* = 8.13kPa *φ* = 10.51°	*c* = 12.08kPa *φ* = 9.61°
		*Fc* = 1.69 *Fφ* = 1.3	*c* = 7.35kPa *φ* = 9.99°	*c* = 6.65kPa *φ* = 7.15°	*c* = 6.92kPa *φ* = 9.72°	*c* = 10.3kPa *φ* = 8.88°
		*Fc* = 1.96 *Fφ* = 1.4	*c* = 6.29kPa *φ* = 9.29°	*c* = 5.65kPa *φ* = 6.64°	*c* = 5.85kPa *φ* = 9.03°	*c* = 8.7kPa *φ* = 8.25°
Double Strength Reduction Method 2		*Fφ* = 1.1	*c* = 10.16kPa *φ* = 11.76°	*c* = 9.23kPa *φ* = 8.43°	*c* = 9.52kPa *φ* = 11.44°	*c* = 14.17kPa *φ* = 10.46°
		*Fφ* = 1.2	*c* = 8.21kPa *φ* = 10.81°	*c* = 7.51kPa *φ* = 7.74°	*c* = 7.69kPa *φ* = 10.51°	*c* = 11.49kPa *φ* = 9.61°
		*Fφ* = 1.3	*c* = 6.52kPa *φ* = 9.99°	*c* = 6.03kPa *φ* = 7.15°	*c* = 6.14kPa *φ* = 9.72°	*c* = 9.18kPa *φ* = 8.88°
		*Fφ* = 1.4	*c* = 5.09kPa *φ* = 9.29°	*c* = 4.76kPa *φ* = 6.64°	*c* = 4.78kPa *φ* = 9.03°	*c* = 7.18kPa *φ* = 8.25°
Double Strength Reduction Method 3	Global Zone	*Fc* = 1.21 *Fφ* = 1.1	*c* = 10.33kPa *φ* = 11.76°	*c* = 9.34kPa *φ* = 8.43°	*c* = 9.67kPa *φ* = 11.44°	*c* = 14.38kPa *φ* = 10.46°
	Shear Zone	*Fφ* = 1.1	*c* = 8.02kPa *φ* = 10.72°	*c* = 7.33kPa *φ* = 7.67°	*c* = 7.51kPa *φ* = 10.42°	*c* = 11.23kPa *φ* = 9.53°
	Global Zone	*Fc* = 1.44 *Fφ* = 1.2	*c* = 8.68kPa *φ* = 10.81°	*c* = 7.85kPa *φ* = 7.74°	*c* = 8.13kPa *φ* = 10.51°	*c* = 12.08kPa *φ* = 9.61°
	Shear Zone	*Fφ* = 1.1	*c* = 6.52kPa *φ* = 9.85°	*c* = 5.92kPa *φ* = 7.04°	*c* = 6.11kPa *φ* = 9.57°	*c* = 9.13kPa *φ* = 8.75°
	Global Zone	*Fc* = 1.69 *Fφ* = 1.3	*c* = 7.35kPa *φ* = 9.99°	*c* = 6.65kPa *φ* = 7.15°	*c* = 6.92kPa *φ* = 9.72°	*c* = 10.3kPa *φ* = 8.88°
	Shear Zone	*Fφ* = 1.1	*c* = 5.08kPa *φ* = 9.1°	*c* = 4.75kPa *φ* = 6.51°	*c* = 4.77kPa *φ* = 8.85°	*c* = 7.15kPa *φ* = 8.08°

**Fig. 9:**
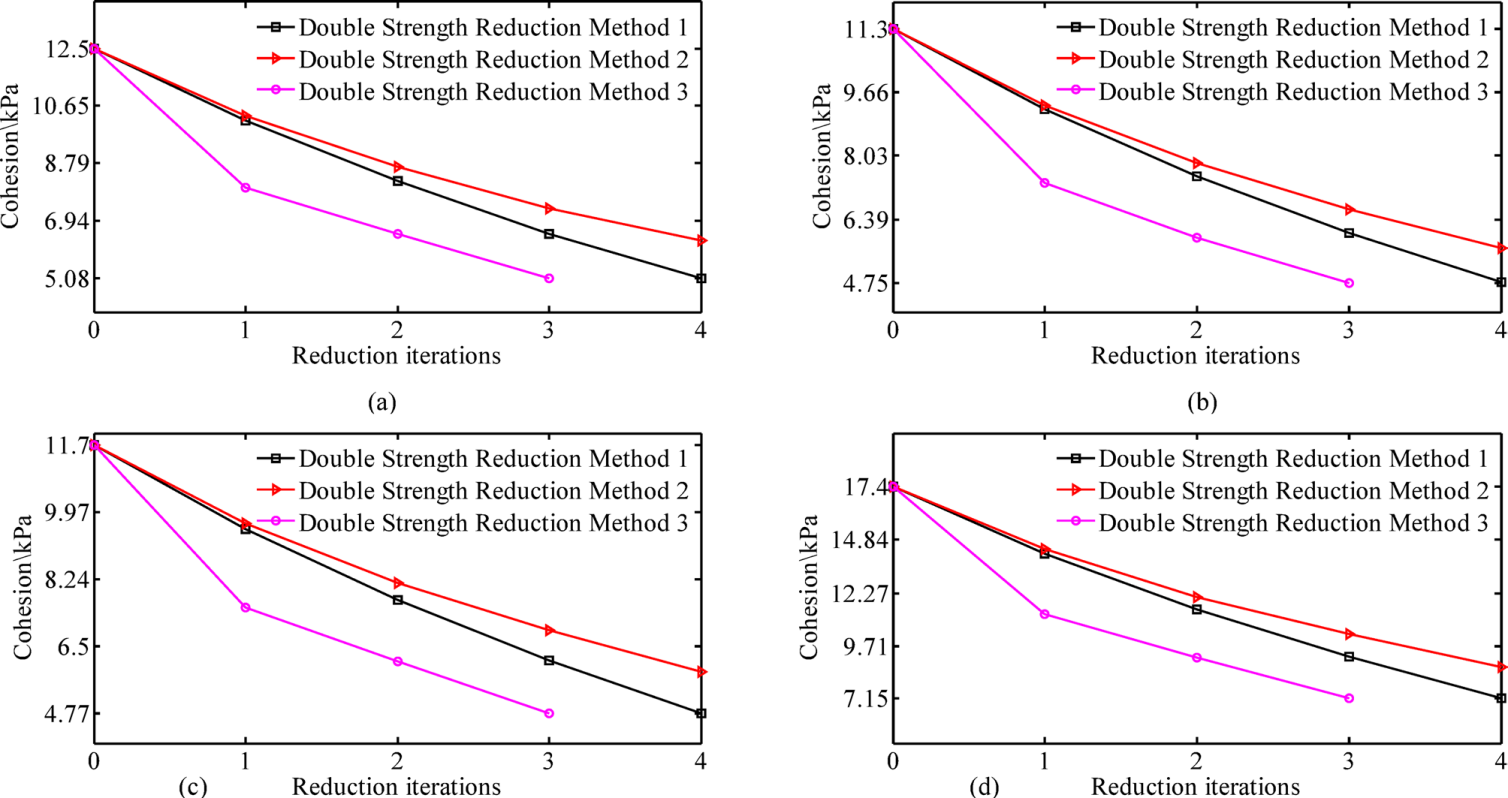
Reduction of soil strength parameters using three double strength reduction methods: **(a)** first layer; **(b)** second layer; **(c)** third layer; **(d)** fourth layer.

**Fig. 10:**
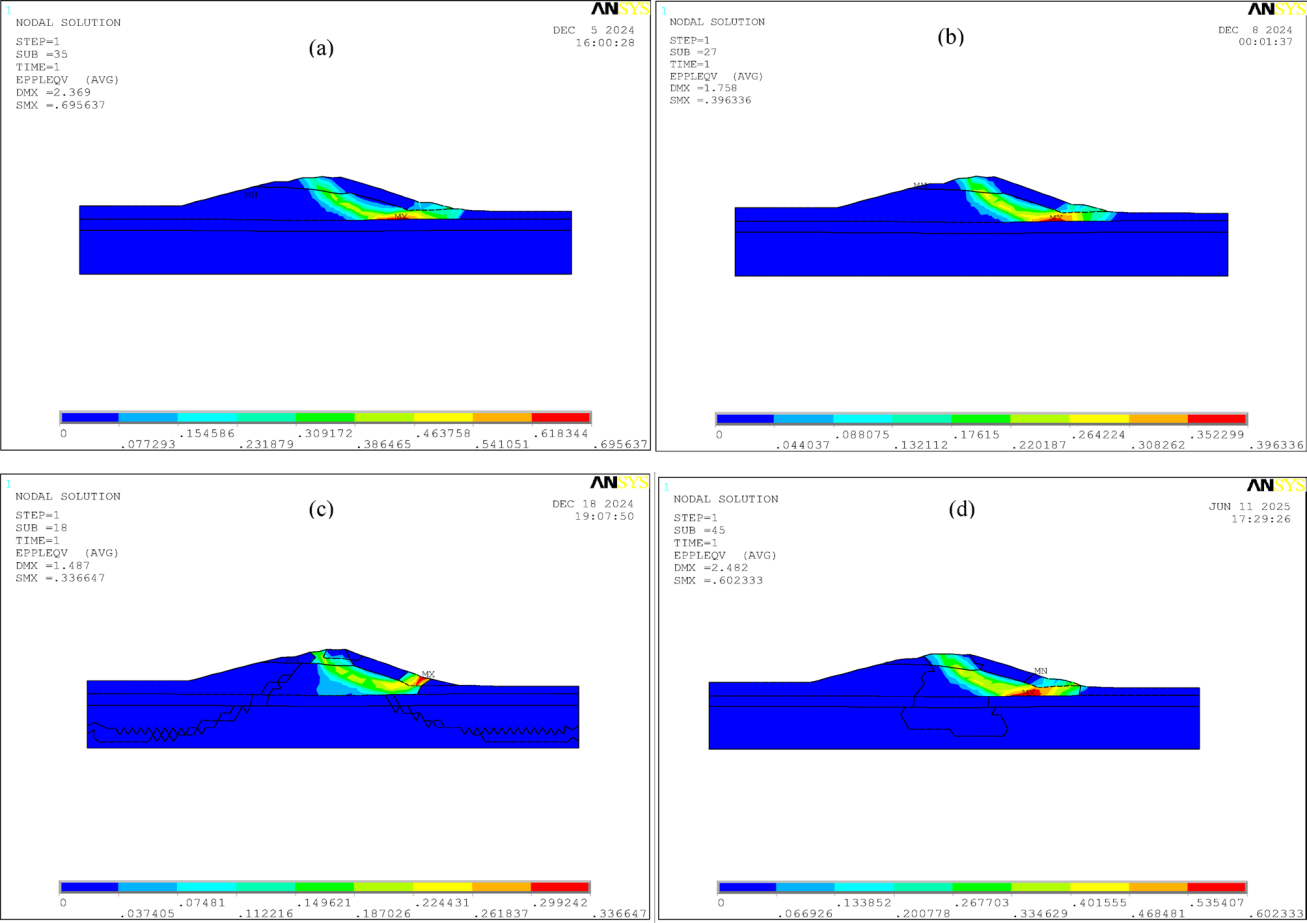
Comparison of critical state plastic zones of Longquan Reservoir Dam using three double strength reduction methods: **(a)** traditional strength reduction method (*Fc* = *Fφ* = 1.8); **(b)** the double strength reduction method 1 (*Fc* = 1.96, *Fφ* = 1.4); **(c)** the double strength reduction method 2 (*Fφ* = 1.4); **(d)** the double strength reduction method 3 (global zone *Fc* = 1.69, *Fφ* = 1.3, shear zone *Fφ* = 1.1).

As shown in Fig. 9; Table 5, the strength reduction coefficients required to reach the critical instability state using different methods exceed 1.0, confirming that the Longquan Reservoir Dam remains in a globally safe state, which is consistent with field observations. During the reduction process of strength parameters, using the double strength reduction method 3 results in the minimum critical strength parameters for each soil layer, and it requires the fewest reduction iterations to reach these critical state parameters(Fig. 9), indicating that this method has the highest computational efficiency. The double strength reduction method 1 yields the largest displacements, stresses, and plastic zones (Fig. 10), while the double strength reduction method 2 yields the smallest values, the double strength reduction method 3 intermediate results. During the deformation evolution process of the dam, the strength parameters of the soil at various locations of the dam will experience varying degrees of degradation. The double strength reduction method 2, which only considers the reduction of shear zone strength parameters, leads to an underestimation of the results. The traditional strength reduction method, which assumes a uniform reduction rate, results in an overestimation. Similarly, the double strength reduction method 1 does not differentiate between the attenuation degrees of shear zone and other areas, leading to an overestimation of the results as well.

**Table 6:** Displacement and stress at monitoring points of the dam.

Monitoring points	Vertical Displacement/cm		Horizontal Displacement/cm		Mises Stress/Pa			
	Node 3	Node 4	Node 6	Node 8	Node 3	Node 4	Node 6	Node 8
Natural State	−41.3	−50.1	6.24	5	17,088	36,580	33,015	34,229
First Reduction	−47.5	−65.9	13.6	8.2	15,384	30,273	33,004	29,048
Second Reduction	−49.5	−99.9	57.9	10.8	16,465	20,384	21,614	23,867
Third Reduction	−47.8	−194.6	196.7	42.4	14,293	11,155	16,154	15,809

**Fig. 11:**
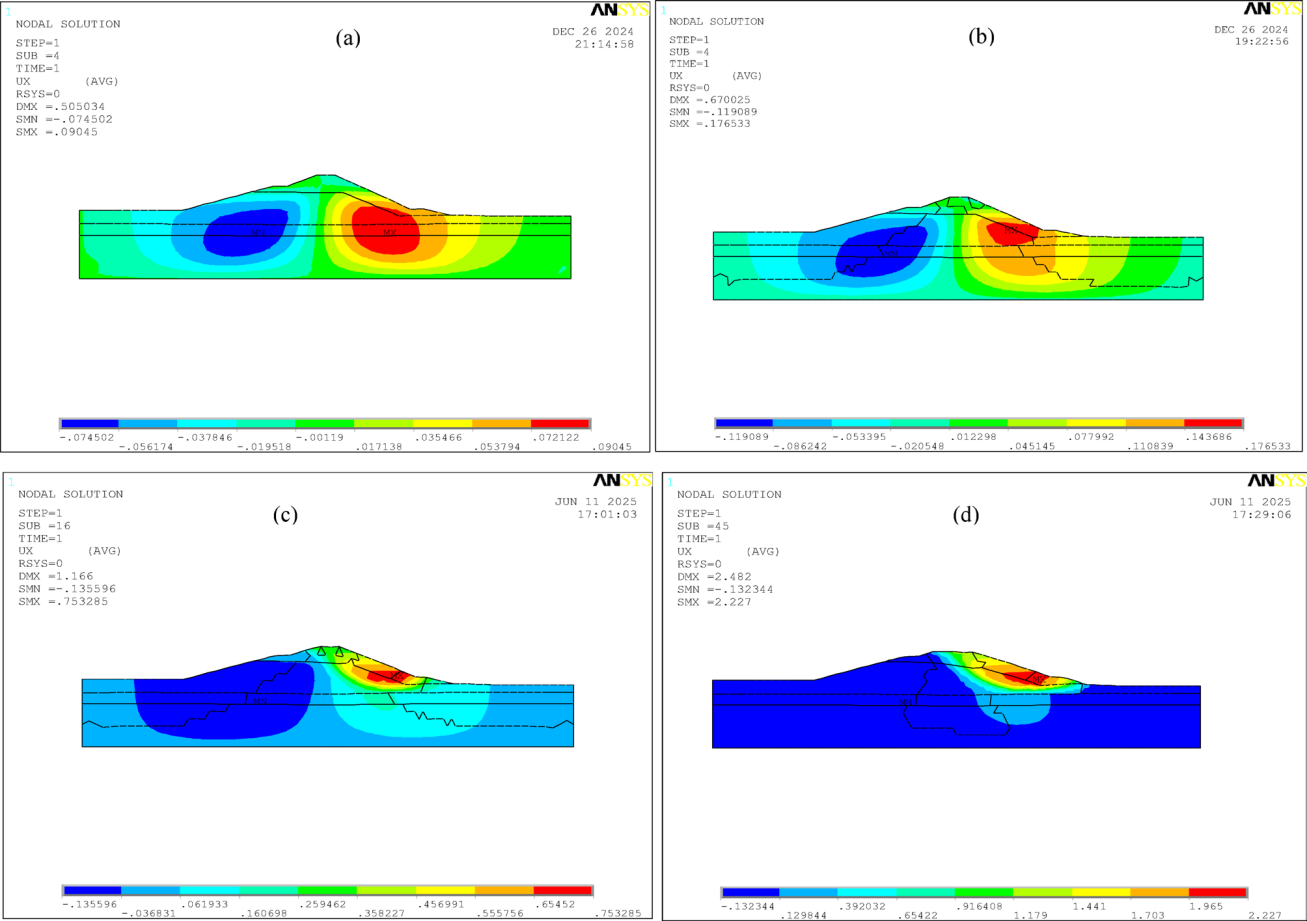
Evolution of horizontal displacement contour maps obtained by the double strength reduction method 3 with the degradation of strength parameters (units: m): (a) original strength parameters; (b) global zone *Fc* = 1.21, *Fφ* = 1.1, shear zone *Fφ* = 1.1; (c) global zone *Fc* = 1.44, *Fφ* = 1.2, shear zone *Fφ* = 1.1; (d) global zone *Fc* = 1.69, *Fφ* = 1.3, shear zone *Fφ* = 1.1.

**Fig. 12:**
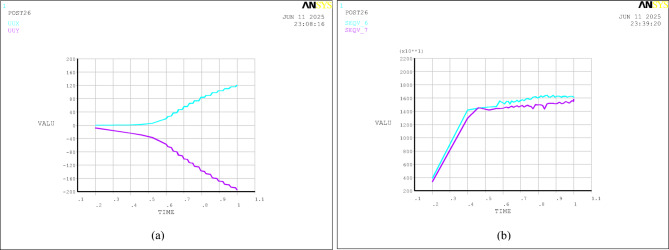
History curves of monitoring points obtained by the double strength reduction method 3 (global zone *Fc* = 1.69, *Fφ* = 1.3, shear zone *Fφ* = 1.1): **(a)** displacement (units: cm); **(b)** stress (units: Pa); Note:UUX denotes the horizontal displacement of Node 4; UUY denotes the vertical displacement of Node 4; SEQV_6 denotes the Mises equivalent stress of Node 6; SEQV_7 denotes the Mises equivalent stress of Node 8.

As shown in Figs. 11 and 12; Table 6, as the strength parameters gradually weaken, the displacements of the monitoring points on the dam generally exhibit a continuously increasing trend. Under self-weight, the dam body will experience vertical settlement. Simultaneously, due to the Poisson effect, the dam body tends to expand horizontally. However, this horizontal expansion is severely constrained by the strong restraining effect from the foundation (valley floor and abutments). Consequently, uplift displacement occurs along the upstream-downstream direction of the dam body in the initial stage of strength parameter degradation. With the continuous weakening of the strength parameters, the upstream uplift deformation gradually evolves into subsidence deformation until the dam crest collapses and merges with the shear zone at the dam base, forming a continuous shear zone. Node 4 is located at the collapsed part upstream of the dam crest, and Node 6 is located at the shear outlet of the shear zone downstream of the dam toe. After the third reduction, the vertical displacement at the dam crest and the horizontal displacement at the dam toe increase significantly, indicating severe collapse at the dam crest and shear sliding at the dam toe, at which point the local stability of the dam body has already failed. Node 8 is located near the shear outlet of the shear zone downstream of the dam toe. During the shear flow process of the soil, the Mises equivalent stress gradually increases (Fig. 12).

**Fig. 13:**
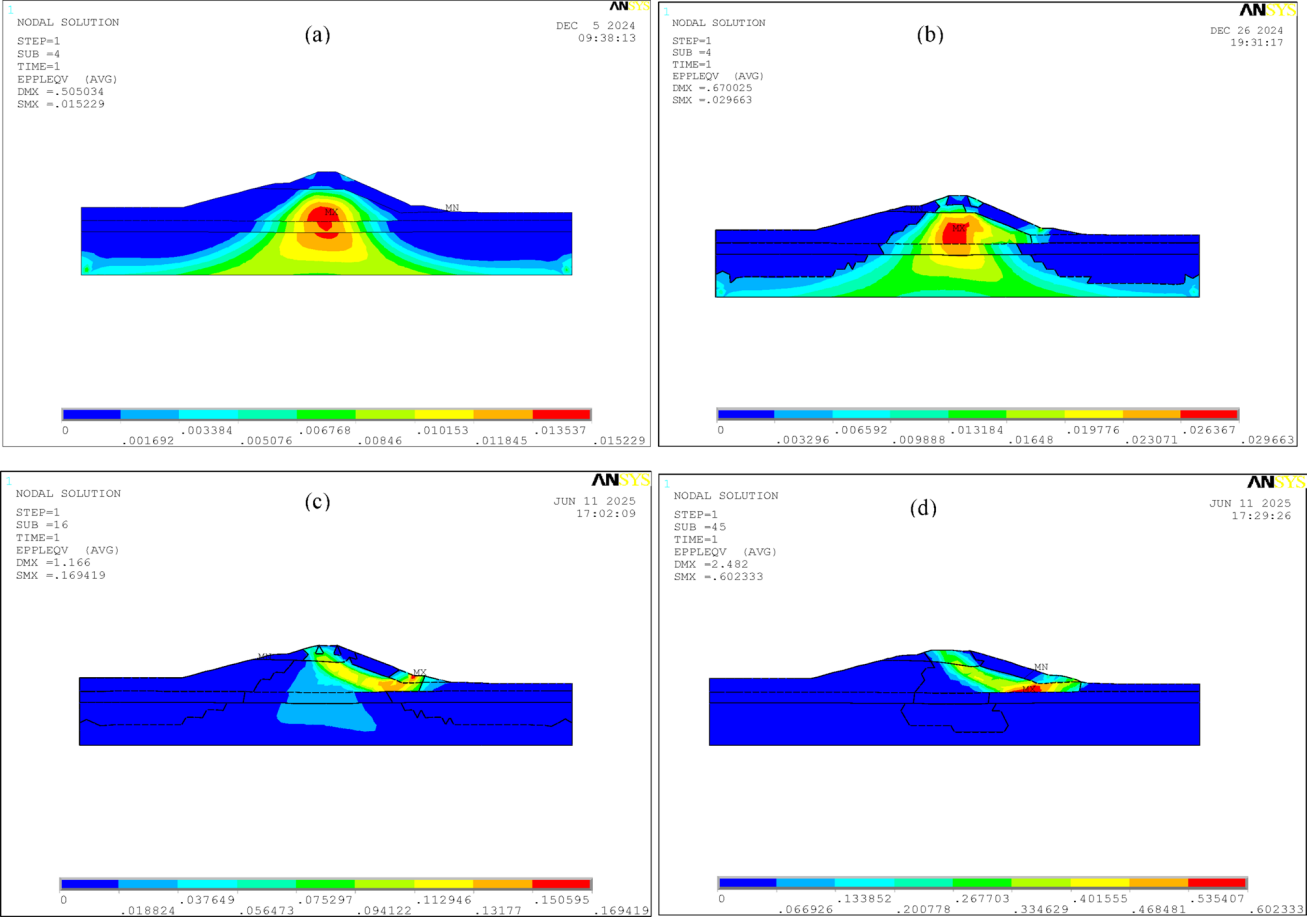
Evolution of plastic strain contour maps obtained by the double strength reduction method 3 with the degradation of strength parameters: **(a)** original strength parameters; **(b)** global zone *Fc* = 1.21, *Fφ* = 1.1, shear zone *Fφ* = 1.1; **(c)** global zone *Fc* = 1.44, *Fφ* = 1.2, shear zone *Fφ* = 1.1; **(d)** global zone *Fc* = 1.69, *Fφ* = 1.3, shear zone *Fφ* = 1.1.

As shown in Fig. 13, with the gradual degradation of the strength parameters, the plastic strain of the dam continues to increase, and the extent of the plastic zone changes, eventually forming a continuous shear zone from the dam crest to the downstream of the dam. Under self-weight, uplift displacements occur both upstream and downstream of the dam, leading to stress release in the soil at the dam toe. After the reduction of strength parameters, plastic yielding and shear flow occur in the downstream soil, resulting in the upstream soil of the dam moving downstream. As the strength parameters continue to weaken, the shear failure zone downstream of the dam toe expands progressively, forming a shear outlet at the dam toe, and the soil in the middle and upper parts gradually evolves into a continuous shear zone extending to the dam crest.

### Analysis of the local failure mechanism of the dam

Under self-weight, uplift displacements occur both upstream and downstream of the dam body, leading to stress relief in the soil at the dam toe. Due to the erosion of the soil by heavy rain, its strength parameters decrease, causing the soil downstream of the dam toe to undergo plastic yielding and shear flow. This leads to the transfer of upstream soil toward the downstream area, gradually forming a subsidence displacement upstream of the dam crest. As the strength parameters continue to weaken, the shear failure zone downstream of the dam toe expands progressively, forming a shear outlet at the dam site until the dam crest collapses, creating a continuous shear zone. After the rain stops, the water level gradually decreases, reducing the lateral pressure on the soil, which causes some parts of the soil to rebound rapidly while others experience delayed rebound. The inconsistent rebound rates of soil deformation lead to the formation of vertical tensile cracks at certain locations, which is one of the important reasons for the cracks in the Longquan Reservoir Dam. Additionally, the stress field in the soil undergoes a sharp shift during heavy rainfall and redistributes after the rain stops. The repeated alteration in the soil stress field exacerbates the development of cracks. Furthermore, vehicles and pedestrians passing over the dam also accelerate the development of cracks.

## Conclusions

Given that the rate and extent of degradation of the soil vary slightly across different parts of the slope (dam), this study proposes an innovative double strength reduction method, the global-local dynamic strength reduction method, which accounts for both the physical degradation laws of soil strength parameters and the strain-softening characteristics of shear zones. The progressive local failure process of Longquan Reservoir Dam under heavy rain conditions was simulated combined with the proposed method, and the dam’s local failure mechanism was revealed through comprehensive analysis of displacement fields, stress distributions, and plastic strain evolution patterns. Additionally, this paper draws the following important conclusions:

(1) All critical strength reduction coefficients exceed 1.0 across different analysis methods, confirming the Longquan Reservoir Dam’s global stability, which is consistent with field observations.

(2) The global-local dynamic strength reduction method can simultaneously capture the physical degradation laws of the strength parameters of soil and the softening mechanism of shear bands, which can more accurately reveal the failure mechanism of the dam. Additionally, the proposed method has high computational efficiency.

(3) Heavy rainfall triggers a progressive local failure process of Longquan Reservoir dam, characterized by two distinct phases: (a) rainfall-induced weakening causes significant reduction in soil shear strength, initiating downstream plastic yielding and upstream soil migration accompanied by progressive crest subsidence; (b) shear failure progression manifests through downstream shear zone expansion and shear opening formation until complete shear band connectivity is established.

(4) The post-rainfall response is characterized by a declining phreatic surface and differential soil rebound. The resulting deformation incompatibility drives the formation of vertical tensile cracks, representing the primary crack initiation mechanism.

It is important to note that the global-local dynamic strength reduction method just estimates rainfall effects through static strength reduction. Although the research outcomes provide important numerical evidence for elucidating the spatiotemporal evolution patterns of rainfall-induced soil strength parameter degradation and the resulting progressive failure mechanisms in slopes (dams), several research extensions remain imperative. First, further validation through systematic in-situ monitoring and laboratory model tests is required to verify the conclusions and promote the practical application of this method in engineering practice. Furthermore, future work should incorporate three-dimensional terrain modeling to better capture geometric complexities in slope (dam) stability analyses.

## Data Availability

All data and material generated or analyzed in this study are included in this manuscript.
